# Phytochelatin Synthase: An In Silico Comparative Analysis in Cyanobacteria and Eukaryotic Microalgae

**DOI:** 10.3390/plants13152165

**Published:** 2024-08-05

**Authors:** Michele Ferrari, Matteo Marieschi, Radiana Cozza, Anna Torelli

**Affiliations:** 1 Department of Biology, Ecology and Earth Science, University of Calabria, Arcavacata di Rende, 87036 Cosenza, Italy; michele.ferrari@unical.it (M.F.); radiana.cozza@unical.it (R.C.); 2 Department of Chemistry, Life Sciences and Environmental Sustainability, University of Parma, Viale delle Scienze 11/A, 43124 Parma, Italy; matteo.marieschi@unipr.it

**Keywords:** phytochelatin synthase, heavy metal, cyanobacterial PCS, microalgal PCS, bioinformatics

## Abstract

Phytochelatins (PCs) are small cysteine-rich peptides involved in metal detoxification, not genetically encoded but enzymatically synthesized by phytochelatin synthases (PCSs) starting from glutathione. The constitutive PCS expression even in the absence of metal contamination, the wide phylogenetic distribution and the similarity between PCSs and the papain-type cysteine protease catalytic domain suggest a wide range of functions for PCSs. These proteins, widely studied in land plants, have not been fully analyzed in algae and cyanobacteria, although these organisms are the first to cope with heavy-metal stress in aquatic environments and can be exploited for phytoremediation. To fill this gap, we compared the features of the PCS proteins of different cyanobacterial and algal taxa by phylogenetic linkage. The analyzed sequences fall into two main, already known groups of PCS-like proteins. Contrary to previous assumptions, they are not classed as prokaryotic and eukaryotic sequences, but rather as sequences characterized by the alternative presence of asparagine and aspartic/glutamic acid residues in proximity of the catalytic cysteine. The presence of these enzymes with peculiar features suggests differences in their post-translational regulation related to cell/environmental requirements or different cell functions rather than to differences due to their belonging to different phylogenetic taxa.

## 1. Introduction

Metal pollution represents a serious concern for the environment and mostly affects aquatic ecosystems. Recently, different studies, on phycodepuration and reviewed by Danouche et al. (2021) [1] and Chakravorty et al. (2023) [2], have focused on heavy-metal algal tolerance and have highlighted that heavy-metal resistance in microalgae can be mediated by several mechanisms varying among different algal strains and depending on the metal kind. These mechanisms include exclusion through binding to the cell wall or low plasma membrane permeability, active extrusion, biotransformation, compartmentalization of heavy metals (HMs) into vacuoles and other intracellular organelles and complexation with chelating agents such as non-proteinaceous compounds (such as malate, citrate, ascorbate and polyphosphates) or metal-binding proteins such as metallothioneins and phytochelatins (PCs) [1,2]. An important role in metal detoxification is played by sulfate metabolism, whose final products are cysteine, glutathione and the molecules derived from them: PCs. PCs [3,4] are cysteine-rich metal-binding peptides with the general structure (γ-Glu-Cys)*n*-Gly (*n* = 2–11) [5], albeit in some plants, the *C*-terminal Gly can be replaced by serine, glutamine, glutamate or alanine [6]. These peptides in plants are enzymatically synthesized from reduced glutathione (GSH) by the enzymes phytochelatin synthases (PCSs) [7,8], enzymes with γ-glutamylcysteine dipeptidyl transpeptidase activity belonging to the same superfamily (PF05023) of papain-like cysteine peptidase [5,9,10]. Since the mid-1980s, studies on the PCSs of various organisms have multiplied and have discovered that these enzymes are diffuse in all eukaryotic kingdoms (plants, algae, fungi and metazoan) [11,12,13,14,15,16,17,18,19]. Moreover, PCS-like proteins are present in several prokaryotes [20,21,22,23]. Differences have been reported between eukaryotic and prokaryotic PCSs [21,24]. The prokaryotic sequences were previously described as half PCS or PCS-like proteins [25], and horizontal gene transfer has been suggested to explain the presence of “prokaryotic” sequences in extremophile green algae [26]. The analysis of PCSs in different plant species indicates that these enzymes contain a highly conserved *N*-terminal domain and a less conserved *C*-terminal domain. In all the known PCSs, the *N*-terminal domain has been reported to confer PCS activity and contains three highly conserved residues corresponding to the Cys_56_, His_162_ and Asp_180_ of *Arabidopsis thaliana* AtPCS1 and acting as a catalytic triad. Site-directed mutagenesis has demonstrated that the substitution of these residues results in the complete loss of AtPSC1 activity [5,8,9]. Prokaryotic sequences mostly refer to the largely studied NsPCS of *Nostoc* sp. PCC7_120_ [20,21]. They have been described as sequences shorter than plant PCSs, containing the *N*-terminal catalytic domain but lacking both the variable *C*-terminal domain and four cysteine residues arranged in CC and CXXXC motifs corresponding to the Cys_90_, Cys_91_, Cys_109_ and Cys_113_ of AtPCS1 [20,21].

Given the catalytic function of the *N*-terminal domain, the function of the *C*-terminal domain has long been debated, given its absence in the PCSs described in prokaryotes. Since plant enzymes are activated by different metal ions, especially bivalent cations, the variable *C*-terminal site of eukaryotic proteins has been proposed to be involved in heavy-metal sensing and -binding via its numerous conserved Cys residues allowing for their translocation to the catalytic *N*-terminal domain [3] and/or in the correct protein folding [27,28,29]. Many studies have indicated that the *C*-terminal domain improves protein stability and enhances PCS activity toward a broad heavy-metal spectrum [5,10,21,30,31]. Metal ions are supposed to directly interact with the Cys-rich *C*-terminal domain [32] or with the GSH substrate through the formation of a metal thiolate [27,28,29]. It is assumed that the synthesis of phytochelatins by PCSs occurs through a two-step ping-pong reaction involving the interaction between two substrates: GSH and the abovementioned thiolate. PCS enzymes are constitutively expressed and subject both to transcriptional regulation through alternative splicing [26,33,34,35] and to post-translational regulation through phosphorylation [27,29]. Studies comparing AtPCS1 and NsPCS demonstrated that metal ions are crucial for a protein conformational change leading to the exposure of a Thr residue, making it available to phosphorylation and giving rise to the creation of a pocket required for the second substrate binding, allowing for the production of longer PC products [27,29]. The lack of the *C*-terminal domain and the phosphorylatable Thr residue are likely at the basis of the reduced metal sensitivity of NsPCS and of its ability to synthesize a product with a low degree of polymerization (PC_2_); this hypothesis was confirmed when it was found that the truncated AtPCS1221 containing only the *N*-terminal domain was able to synthesize PCs to the same extent as AtPCS1 when exposed to Cd, but with a low level of polymerization [21]. The described NsPCS form was thus interpreted as a primitive progenitor form of eukaryotic PCSs mainly involved in GSH homeostasis/metabolism rather than in PC production [21].

PCSs are constitutively expressed, apparently in an inactive form, even in the absence of metal exposure. Furthermore, in some microorganisms expressing the gene, phytochelatins have never been detected. Altogether these observations led to the hypothesis that PCSs not only intervene in heavy-metal detoxification [8,36,37,38,39] but also play a preeminent role in essential metal homeostasis, in GS-conjugate metabolism and in GSH homeostasis and immune response [4,22,25,40,41,42,43,44].

Due to the complexity of their regulation, the PCS action mechanism is, as yet, not fully understood.

In a recent paper by Filiz and coworkers (2019) [45] the differences in the PCSs of various higher plants were analyzed, but a similar study has never been performed in algae. Given that heavy-metal pollution strongly impacts the aquatic environment, we considered it very important to analyze the sequences of PCSs in different aquatic organisms in an attempt to give more insights in the evolution of PCS proteins, responsible for the biosynthesis of one of the main heavy-metal detoxifying peptides—PC—in plants. Our work indicates that both eukaryotic algae and cyanobacteria possess more than one isoform of PCSs, or PCS-like proteins and forms with or without a *C*-terminal domain; this suggests that contrary to previous assumptions, the main differences were not due to the divergence between prokaryotic and eukaryotic enzymes but rather to different cell/environmental requirements or different cell functions.

## 2. Results and Discussion

### 2.1. PCS Phylogenetic Analysis

The known *Chlamydomonas reinhardtii* PCS amino acid sequence, Cre07.g319500, from the annotated genome of this alga [46,47] in the Phytozome database [48] was compared with the two model PCS sequences described for land plants (AtPCS1 of *A. thaliana*) and for prokaryotes (NsPCS AD1928 of *Nostoc* sp. PCC7_120_) [20]. The alignment among AtPCS1, Cre07.g319500 and NsPCS AD1928 (now replaced by Q8YY76_ANASP) is shown in Figure S1. The *C. reinhardtii* PCS shares many features with AtPCS; beside the *N*-terminal conserved region corresponding to the phytochelatin domain (EC 2.3.2.15), it indeed possesses a *C*-terminal variable region rich in cysteine residues and the four conserved cysteines described in land plant PCSs [20,21]. The Cre07.g319500 PCS was used as a reference in NCBI data banks to retrieve more than 200 sequences from different cyanobacteria and algae belonging to different taxa (Table S1). Owing to different representation in data banks, some taxa are probably under-represented in this analysis (e.g., brown and red algae).

**Figure 1:**
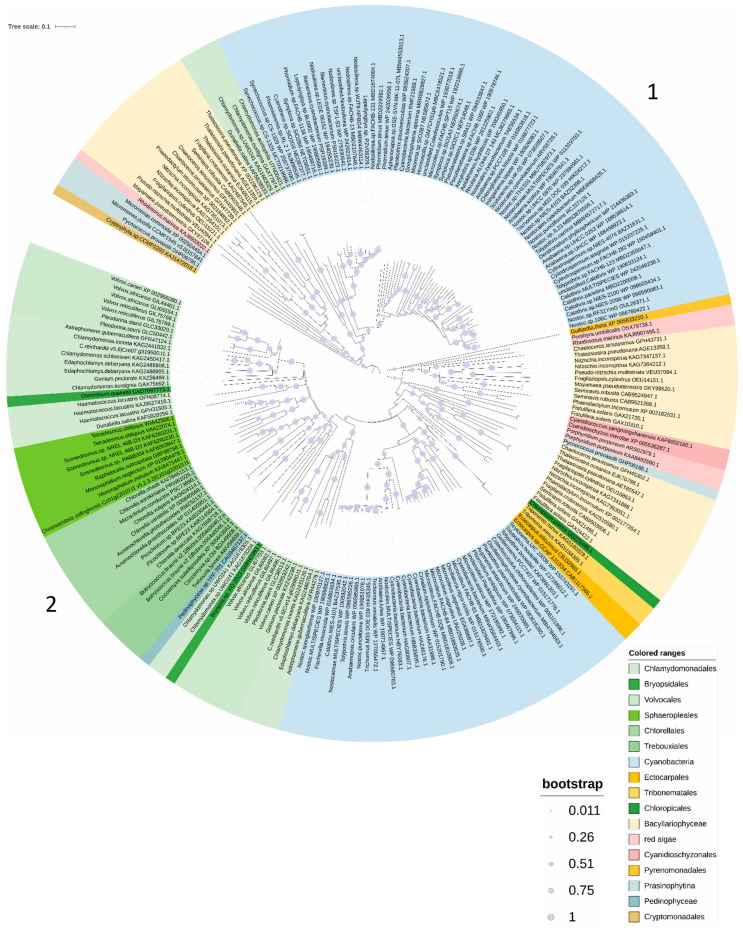
The evolutionary history of PCSs. The maximum likelihood tree is shown. The PCS sequences are split into two main clusters indicated as branches 1 and 2. The PCS sequences are split into two main clusters indicated as branches 1 and 2.The percentage of trees in which the associated taxa clustered together is shown below the branches. The tree is drawn to scale, with branch lengths measured in the number of substitutions per site. This analysis involved 220 amino acid sequences (Table S1) for a total of 3208 positions in the final dataset.

The phylogenetic analysis of PCS and PCS-like sequences, performed through the alignment with homologous sequences retrieved from NCBI data banks, is reported in Figure 1. The PCS sequences are split into two main clusters indicated as branches 1 and 2.

Both branches contain cyanobacterial and algal sequences. In the first branch (group 1), there are cluster sequences with similar features to those previously described for cyanobacterial sequences. They indeed share features with NsPCS, lacking the variable *C*-terminal and the four conserved cysteine residues found in the land plants *N*-terminal conserved domain and corresponding to the Cys_90_, Cys_91_, Cys_109_ and Cys_113_ of AtPCS1 [21,24]. Together with numerous cyanobacteria, in this group, there are two clearly identifiable sub-branches, with one containing the PCS sequences of diatoms, red algae and Prasinophytina and the other containing the sequences of the extremophile Chlorophyceae, more closely related to cyanobacteria than to other eukaryotic algae present in this group. We thus confirmed the presence of “half PCS-like protein”, also described as “primitive PCS”, in eukaryotic extremophile green algae other than those reported by Olsson et al. (2017) supposed as originated from horizontal gene transfer [26]. In the second branch (group 2), there are cluster sequences similar to those previously described for AtPCS1 and land plants [45]; in this group, there are also three sub-branches, where the first two are apparently early-diverging and include different sequences of red algae, diatoms and Prasinophytina and the third includes cyanobacteria, some Ectocarpales and Tribonematales and the majority of green algae. In both groups 1 and 2, no cyanobacterial sequences grouped close to red algae or diatoms, likely because the sequences of the prokaryotic ancestors of the red algal plastids are poorly represented in GenBank.

Differently from what has been reported in previous papers, describing for cyanobacteria only “half PCS-like proteins” [20,21,24,25,26], our observations lead to the important consideration that at least some cyanobacteria (e.g., Nostocales and Oscillatoriales) (Figure S2) possess two kinds of PCSs, one with the features previously described for cyanobacterial short sequences and another closer to land plant PCSs. In both groups 1 and 2, the cyanobacterial sequences are more closely related to the Chlorophyceae, indicating a common evolution, whereas sequences of the green algae Prasinophytina cluster in an independent sub-branch, indicating an independent evolutionary history. Moreover, the red algae of both groups 1 and 2 cluster in a basal branch belonging to the diatom sub-trees, in agreement with the hypothesis of secondary endosymbiosis of the latter based on unicellular red algae.

Within group 2, red algae, diatoms and Chlamydomonadales sequences (among green algae) are further divided into two sub-groups, suggesting that gene duplication occurred within taxa in the Chlamydomonadales and red algae or in the red common ancestor in the case of diatoms.

### 2.2. PCS Sequence Analyses

**Figure 2:**
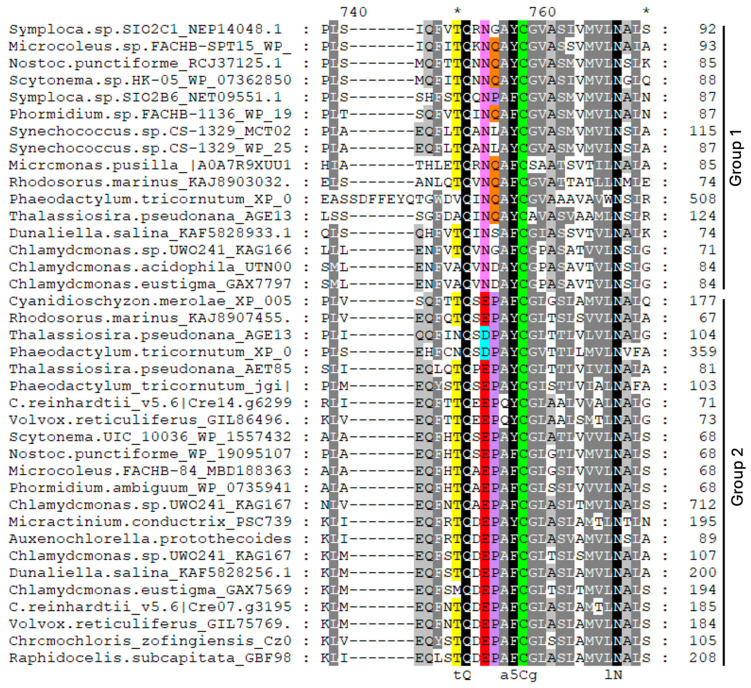
Partial representation of multiple-sequence alignment of PCS proteins showing discriminant residues N, E and D (excerpt of Figure S2). Partial representation of multiple-sequence alignment of PCS protein sequences referred to in Table S1 and Figure S2. The selected sequences were chosen as representatives of different taxa. Alignment was conducted with ClustalW; identical and similar residues are shaded in black and gray, and consensus sequence is shown below alignment. Sequences of group 1 are characterized by one asparagine residue (N, magenta), often followed by a glutamine (Q, orange) four amino acids upstream of the catalytic cysteine (C, green). In the sequences of group 2, the asparagine residue is substituted by residue of glutamic acid (E, red), or by aspartic acid (D, cyan) in a sub-group of diatoms, followed by a proline (P, lilac). In yellow, the threonine (T) residue is a possible target of phosphorylation.

The analysis of the PCS sequences represented in the phylogenetic tree (Figure 1) revealed that all the sequences belonging to group 1 (Figure 2 and Figure S3) are characterized by an asparagine residue (Asn, N, magenta in Figure 2 and Figure S3), often followed by a glutamine (Glu, Q, orange in Figure 2 and Figure S3) four amino acids upstream of the catalytic cysteine (Cys, C, green in Figure 2 and Figure S3). In the sequences of group 2, the Asn residue is substituted by the residue of glutamic acid (Glu, E, red in Figure 2 and Figure S3) or by aspartic acid (Asp, D, cyan in Figure 2 and Figure S3) in a sub-group of diatoms, followed by a proline (Pro, P, lilac in Figure S2). This distribution is extremely interesting, since this amino acid substitution could deeply affect protein conformation, since proline generally confers more rigidity to the secondary structure. The substitution of the Asn residue with Asp or Glu can also play a role in PCS post-translational regulation through phosphorylation. The phosphorylation of a threonine residue (Thr, T) placed upstream of the catalytic cysteine (corresponding to Thr_49_ and Cys_56_ in AtPCS1, respectively) has indeed been described by Wang and coworkers (2009) [27] as an important point for the regulation of PCS activity in A. thaliana. This Thr residue (yellow in Figure 2 and Figure S3) is highly conserved albeit absent in the red algae and diatoms of group 2 and sub-group D of group 1. Notwithstanding this strong conservation only in the PCSs of group 2, the presence of glutamic (E) or aspartic acid (D) ensures the right context ([ST]-X2-[DE]) allowing the Thr residue to be a target of casein kinase 2 (CK2) activity [27]. On the contrary, in the sequences of group 1, this context is lost due to the presence of asparagine (N) instead of D/E, as described for NsPCS [27], which is not subject to phosphorylation.

**Figure 3:**
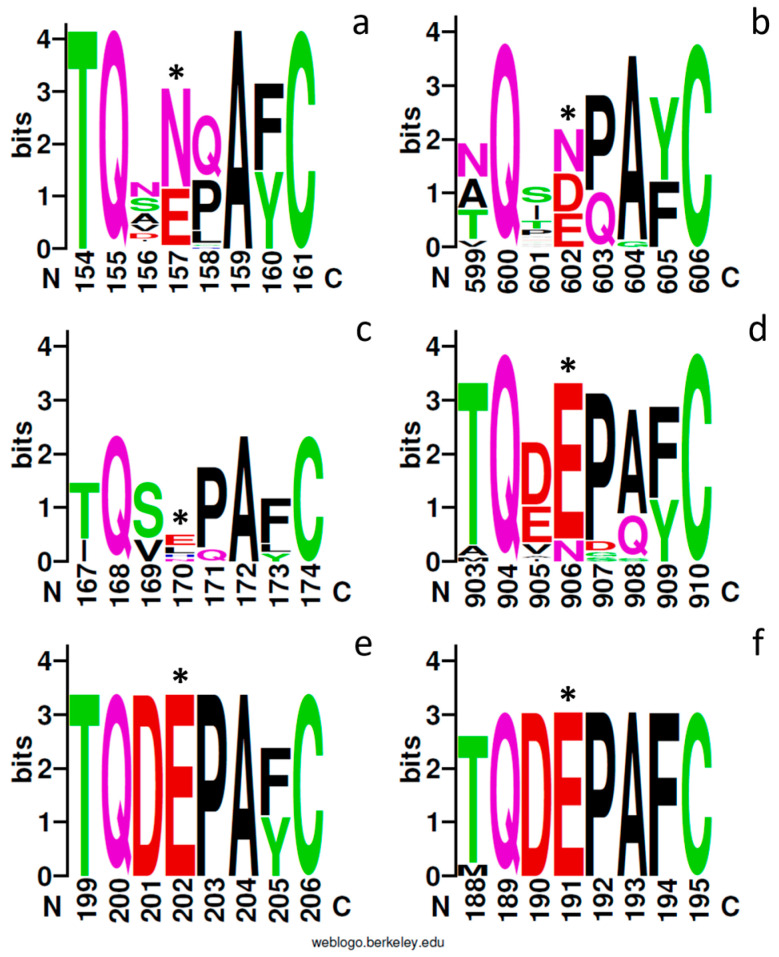
The WebLogo of the sequence preceding the conserved Cys residue of the catalytic triad in different taxa. The sequences start from the initial Thr residue presumably involved in post-translational regulation through phosphorylation and the alternative residues characterizing the “N”, “E” and “D” isoforms (this position is marked with an asterisk). (**a**) Cyanobacteria; (**b**) diatoms; (**c**) red algae; (**d**) Chlamydomonadales; (**e**) Trebouxiophyceae; (**f**) Sphaeropleales.

The WebLogo representation reported in Figure 3 shows the amino acidic context surrounding catalytic cysteine in different taxa.

#### 2.2.1. Cyanobacteria

**Figure 4:**
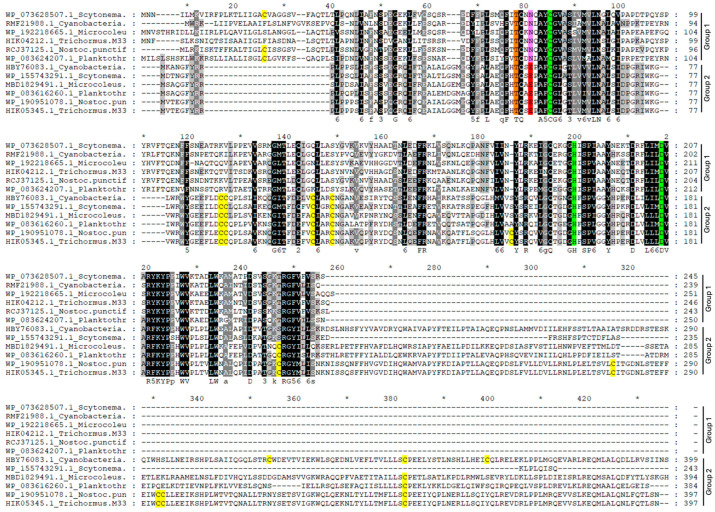
Multiple-sequence alignment of PCS proteins of cyanobacteria possessing both “N” and “E” isoforms. Alignment was conducted with ClustalX 2.0; identical and similar residues are shaded in black and gray, and the consensus sequence is shown below the alignment. Catalytic triad residues (Cys, His and Asp) are highlighted in green and cysteines in yellow. In orange is shown the Thr residue passible of phosphorylation, and in magenta and red are highlighted the Asn and Glu residues characteristics, respectively, of group 1 or 2 in the phylogenetic tree represented in Figure 1.

Cyanobacteria belonging to the Oscillatoriales or Nostocales orders possess two PCS-like sequences characterized by the alternative presence of the couple of residues N or E upstream of the catalytic cysteine (C_161_) (Figure 3a, position marked by an asterisk). As shown in the alignment in Figure 4, both “N” and “E” cyanobacterial sequences possess a threonine residue (orange in Figure 4) seven amino acid residues upstream of the catalytic cysteine; but only in PCS “E”, this is in the right context to be a target for CK2 phosphorylation. As shown in Figure 4, cyanobacterial sequences belonging to group 2 extend at the *C* terminus more than group 1 sequences.

#### 2.2.2. Diatoms

**Figure 5:**
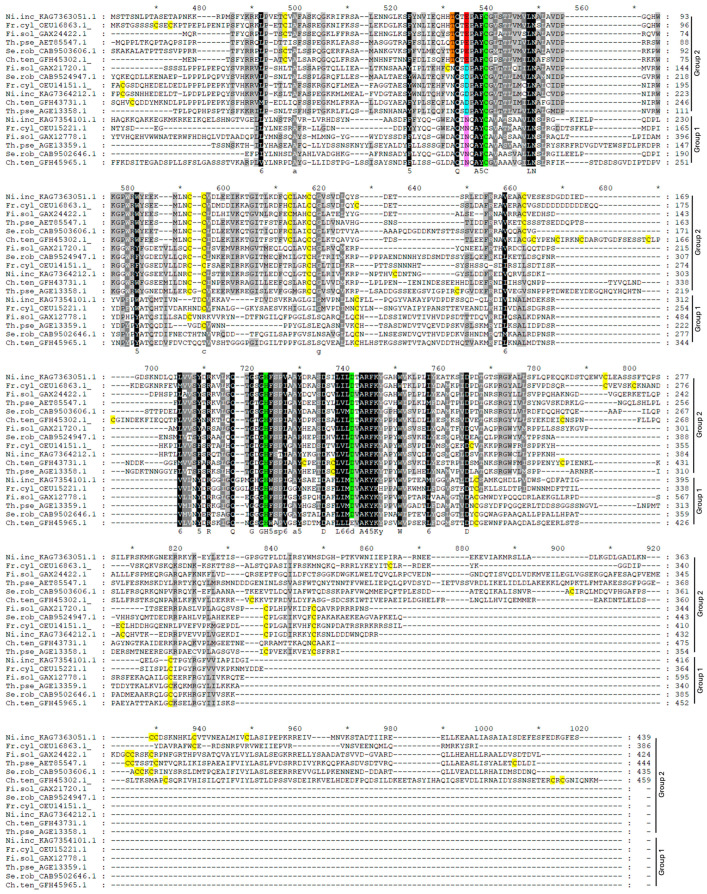
Partial representation of multiple-sequence alignment of PCS proteins of diatoms possessing “N”, “E” and “D” isoforms. Alignment was conducted with ClustalW; identical and similar residues are shaded in black and gray, and consensus sequence is shown below alignment. Catalytic triad residues (Cys, His and Asp) are highlighted in green and cysteines in yellow. In red, cyan and magenta are highlighted the Glu, Asp, (characteristic of sub-branches “E” and “D” of group 2) and Asn residues of group 1, respectively, in the phylogenetic tree in Figure 1. In orange, is indicated the Thr residue, a possible target of phosphorylation.

Diatoms possess three different PCS sequences (as indicated by the presence of the same taxa in three different clusters of the phylogenetic tree), with one belonging to group 1 (N residue in magenta in Figure 5) and two being more closely related to each other and split into two sub-branches belonging to group 2. The latter are characterized by the alternative presence of glutamic acid (E, pale blue in Figure 5) or aspartic acid (D, red in Figure 5) upstream of the catalytic cysteine (logo in Figure 3b and alignment in Figure 5). In these algae, however, the threonine residue (T, orange in Figure 5) described as subject to phosphorylation is conserved only in the sequences belonging to the “E” sub-branch characterized by the presence of glutamic acid nearby catalytic cysteine, which also possess a *C*-terminal domain longer than the sequences of the other diatom sub-branches (Figure 5).

#### 2.2.3. Ocrophyta

For Ocrophyta, other than diatoms, only two PCS sequences were found, and this makes the analysis impossible for this taxonomic group; however, both *Tribonema* sequences are similar to the diatom “D” form, while the *Ectocarpus* sequences belong to the diatom “E” sub-branch. In all these sequences, the Thr residue is conserved and in the right context to be a target of CK2 ([ST]-X2-[DE]).

#### 2.2.4. Archaeplastide

##### Red Algae

Few red algal PCS sequences (only seven accessions) were found in data bank mining, and most of them cluster in group 2, but the Stylonematales *Rhodosorus marinus* possesses sequences of both types (“N” and “E”), thus suggesting that two different PCSs are present also in this taxonomic group. The logo of these few sequences is reported in Figure 3c, and only the Cyanidioschizonales and the *R. marinus* belonging to group 2 are likely possible targets of phosphorylation.

##### Green Algae

We found only few PCS accessions for the early diverging Prasinophytina. Among these, for the Mamiellales *Micromonas commoda* and *Micromonas pusilla*, we retrieved only sequences belonging to group 1 and clustering in a branch separated from the remaining green algae (Figure 1). The Pseudoscorfieldiales *Pycnococcus provasolii* is instead present in both groups 1 and 2. As already stated, this discrepancy may represent a real difference between taxa but could also be due to a poor representation of these algae in the databases. Within the Chlorophyceae, the Chlamydomonadales apparently possess two PCS sequences. In most species, both PCS sequences cluster into closely related sub-branches, both belonging to group 2 and distinguishable by the alternative glutamate (E) or aspartate residue (D) preceding the E residue characterizing this branch (Figure 3d). With the exception of *Chlamydomonas eustigma* GAX75692, all these sequences possess the conserved Thr residue in the right context to be the target of phosphorylation (Figure S2). A little group of extremophilic Chlamydomonadales (*Dunaliella salina*, *C. eustigma*, *Chlamydomonas acidophila* and the arctic strain *Chlamydomonas* sp. UWO24) instead possess two PCS sequences, with one clustering in group 1 and the other in group 2, as it occurs in Nostocales and Oscillatoriales cyanobacteria.

In the Trebouxiophyceae, only one PCS form, belonging to group 2, was found, and the presence of more than one accession for the same organism is due to different sequencing projects for the same alga. A similar situation was found in the Sphaeropleales. In this case, however, some duplicate sequences are seemingly due to the presence of two alleles in algae with diploid genomes [49]. In these two latter algal orders, sequences are highly homogeneous, as attested by the uniformity of their logo image (Figure 3e and Figure 3f for the Trebouxiophyceae and Sphaeropleales, respectively), and with the only exception of *Scenedesmus* sp. KAF6260230, all these proteins are likely passible of post-translational regulation through phosphorylation (Figure S2).

### 2.3. PCS Proteins in Selected Model Sequences

We analyzed more in depth the different PCS sequences of some organisms selected as representatives of the different taxa. In this group of organisms, we included six cyanobacteria (Nostocales: *Nostoc punctiforme*, *Scytonema* sp.; Oscillatoriales: *Microcoleus* sp. *Phormidium* spp.; Coleofasciculales: *Symploca* sp. and Synecococcales: *Synecococcus* sp.), two red algae (Stylonematales: *Rhodosorus marinus*; Cyanidiales: *Cyanidioschizon merolae*), two diatoms (Bacillariales: *Phaeodactylum tricornutum* and *Thalassiosira pseudonana*, representatives of Pennales and Centrales diatoms, respectively) and ten green algae (Chlamydomonadales: *C. reinhardtii*, *Chlamydomonas* sp. UWO241, *C. eustigma*, *C. acidophila*, *Volvox reticuliferus* and *D. salina*; Chlorellales: *Auxenochlorella prototecoides* and *Micractinium condutrix*; Sphaeropleales: *Raphidocelis subcapitata* and *Chromochloris zofingiensis*).

As reported in Table 1, the sequences belonging to group 1 and characterized by the Asn residue (N, in bold in Table 1) are generally shorter than their counterparts belonging to group 2, and the *C*-terminal variable domain is lacking (cyanobacteria) or is very short (eukaryotic algae).

The PCS transcripts of diatoms, red algae and *Micromonas*, belonging to the early diverging order of the Mamiellales, are composed by a low (one–three) number of exons, regardless of the isoform, sharing more similarity with the intron-less cyanobacterial sequences, with the only exception of the isoform of *R. marinus* clustering in group 2, which is composed by five exons. Green algae have instead evolved a multi-exon organization in their transcripts, even in the sub-group of the extremophilic Chlamydomonadales PCS sequences clustering in group 1.

**Figure 6:**
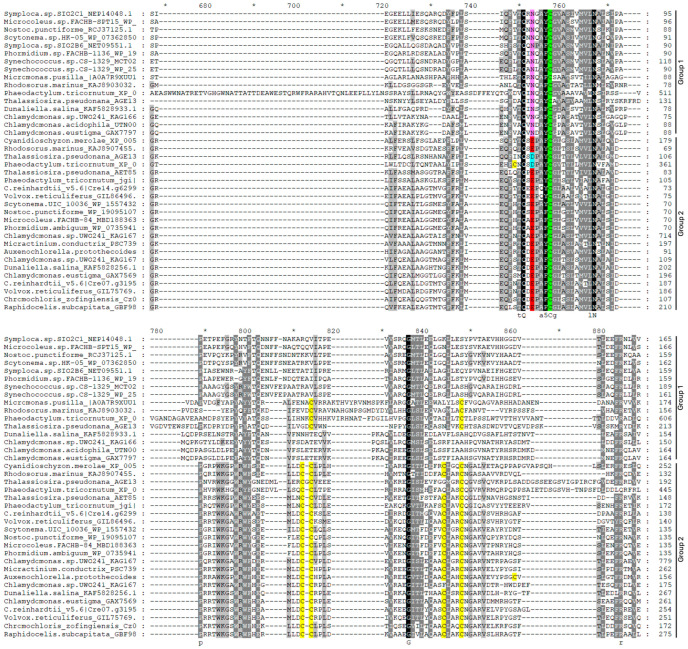
Partial representation of multiple-sequence alignment of PCS proteins of the model PCS sequences referred to in Table 1. Alignment was conducted with ClustalX 2.0; identical and similar residues are shaded in black and gray, and consensus sequence is shown below alignment. Cys residues of the catalytic triad (Cys, His and Asp) is highlighted in green, and other conserved cysteines are highlighted in yellow. Asp, Glu (characteristic of the sub-branches “D” and “E” of cluster 2) and Asn residues of cluster 1 of the phylogenetic tree in Figure 1 are highlighted in red, cyan and magenta, respectively.

Both the cyanobacterial and eukaryotic sequences of group 1 are poorer in Cys residues (1–3 for cyanobacteria and 1–7 for eukaryotic algae) than the sequences of group 2 (6–11 for cyanobacteria and 9–32 for eukaryotic algae). In both groups, the distance between catalytic Cys and His is more variable than the distance between His and Asp of the catalytic triad, which is greatly stable (17 residues). Moreover, the sequences belonging to the different sub-branches show different cysteine arrangements regarding the four conserved cysteines described for land plant PCSs [24]. These conserved cysteines, corresponding to Cys_90_, Cys_91_, Cys_109_ and Cys_113_ of *At*PCS1, are placed between catalytic Cys and His and arranged into two groups (CC and CXXXC) separated by nearly 20 residues in the group 1 “E” branch, whereas only three of them are present in the diatoms “D” sub-branch sequences. The presence/absence of the four conserved Cys residues described for *At*PCS1 is reported in the alignment of the PCS sequences of the selected organisms shown in Figure 6 (Cys residues highlighted in yellow). In group 1, the Mamiellales, diatoms and *Rhodosorus* conserved two of these cysteines, the first of the motif CC and the second of the motif CXXXC, whereas the extremophilic Chlamydomonadales sequences are more similar to those of cyanobacteria lacking all these four cysteines. This observation supports a common origin of Chlorophycean and cyanobacterial PCS-like proteins separated from other Archaeplastida enclosed in group 1.

Differences among PCSs were also observed in various protein parameters analyzed with the ProtParam tool [50] (also reported in Table 1). No significant differences between proteins of groups 1 and 2 were observed as regards the Isoelectric Point nor their percentage of negative or positive residues. Cyanobacterial PCSs (regardless of their belonging to group 1 or 2) showed a higher aliphatic index (*p* < 0.01) than those of eukaryotic algae; this should be interpreted as an index of higher thermostability of the corresponding proteins. Albeit, in eukaryotic PCSs, the *C*-terminal domain has been predicted to stabilize the protein [31], in both cyanobacterial and eukaryotic sequences, the PCS isoforms of group 1 (lacking the *C*-terminal domain) are indicated as more stable (*p* < 0.01) than the proteins belonging to group 2 (instability index higher than 40). This predicted higher stability is maybe the reason for the retention of these isoforms in eukaryotic algae living in extreme environments, whereas in freshwater algae, the isoforms of group 2 (“E”) prevail. Despite the multi-exon organization indicating a separate evolution, the sequences of the extremophilic Chlorophyceae are closer to the “N” isoforms of cyanobacteria than are the Mamiellales sequences with regard to other features, such as the organization of a particular group of cysteines (see below).

Unfortunately, the cell localization of algal sequences is still hardly predictable by algorithms trained on higher plants, so we were unable to find and discuss this information.

### 2.4. Structural Analysis

#### 2.4.1. Active Site

**Figure 7:**
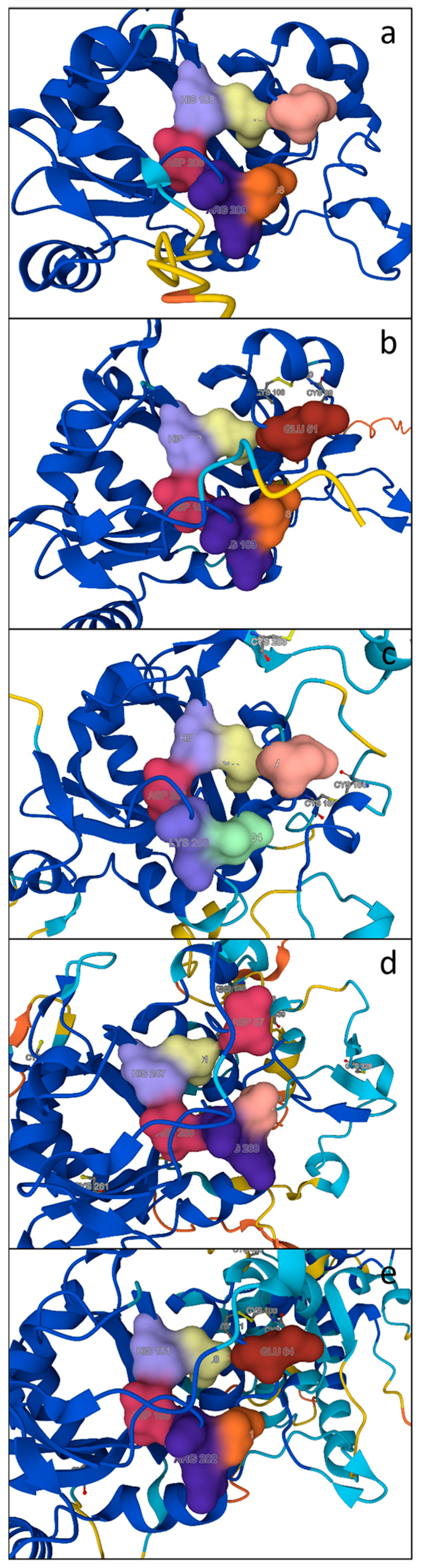
Predicted 3D model of catalytic triad (Cys in yellow, His in lilac and Asp in red) and residues potentially involved in PCS post-translational regulation (Thr in orange, Arg in violet, Glu in dark red and Asp in red) (see text). Alternative residues occupying the same positions are Asn (in pink), Lys (in lilac) and Ala (in green). (**a**) *Scytonema* sp. WP_073628507.1 “N” isoform; (**b**) *Scytonema* sp. WP_155743291.1 “E” isoform; (**c**) *T. pseudonana* AGE13359.1 “N” isoform; (**d**) *T. pseudonana* AGE13358.1 “D” isoform; (**e**) *T. pseudonana* Thaps_257216 “E” isoform. Models were generated by using Alphafold.

In order to gain further insights into the functional forms of PCS proteins, a structural analysis was performed on the predicted PCS models retrieved from the AlphaFold database [51,52]. In Figure 7, we report the structures of the active site and the residues potentially involved in regulation through phosphorylation in the cyanobacterium *Scytonema* sp. (chosen as an example of cyanobacterium owning sequences belonging to both groups 1 and 2) (Figure 7a and Figure 7b, respectively) and in the diatom *T. pseudonana*, in which all the three representative isoforms of eukaryotic algae, characterized by N, D or E residues four aa upstream of the catalytic cysteine (Figure 7c, Figure 7d and Figure 7e, respectively), have been identified. According to the results of Wang et al. (2009) [27], only *Scytonema* and *Thalassiosira* “E” are apparently putative targets for phosphorylation, being the only two which possess a Thr (orange in Figure 7b,e) residue placed at the right distance from a Glu residue (red in Figure 7). The presence of an Asp residue in diatom “D” should allow for phosphorylation, but in these sequences, the Thr residue (or eventually Ser) is substituted by an Asn residue (pink in Figure 7d) that is not a target of phosphorylation.

The existence of the N and E forms both in cyanobacteria and in eukaryotic algae leads us to rethink what in previous works [21] was indicated as the difference between cyanobacterial and eukaryotic PCSs, with NsPCS being described as the prokaryotic progenitor of eukaryotic PCSs.

Subsequent analyses conducted by Wang et al. (2009) [27] led to the hypothesis that AtPCS1 and NsPCS had different functions due to the metal insensitivity of the latter, its inability to form a binding pocket for the second substrate and consequently its inability to produce PCs with a high degree of polymerization. The authors concluded that prokaryotic NsPCS-like “half PCS sequences” may be more likely involved in GSH metabolism rather than PC production. Our results support this assumption and indicate that further analyses should be conducted on the same “N” or “E” isoform to gain more insights into the real differences between these two kinds of enzymes in prokaryotes and eukaryotes.

#### 2.4.2. Cysteine Arrangements

**Figure 8:**
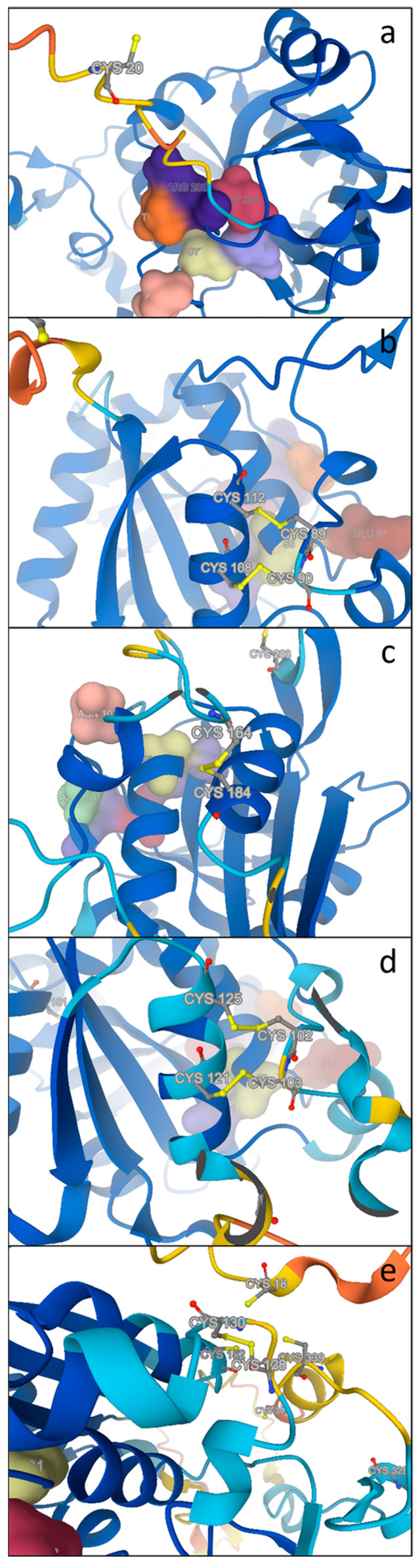
Predicted 3D model showing the different arrangements in the four conserved cysteine residues (represented in ball and stick) described in *At*PCS1 (highlighted in yellow in Figure 7). (**a**) *Scytonema* sp. WP_073628507.1 “N” isoform; (**b**) *Scytonema* sp. WP_155743291.1 “E” isoform; (**c**) *T. pseudonana* AGE13359.1 “N” isoform; (**d**) *T. pseudonana* AGE13358.1 “D” isoform; (**e**) *T. pseudonana* Thaps_257216 “E” isoform. Models were generated by using Alphafold.

The different cysteine arrangements described in Figure 6 give rise to different implications, as shown by structural analysis conducted with Alphafold [51,52] and can contribute to correct protein folding. In Figure 8, we reported the different cysteine arrangements in the cyanobacterium *Scytonema* and in the diatom *T. pseudonana*. The “N” sequence of the cyanobacterium *Scytonema* shows a single cysteine residue in addition to that of the catalytic triad (Figure 8a), whereas in its counterpart “E” PCS sequence, the four cysteine residues of the conserved motifs CC and CxxxC (Cys_89_, Cys_90_, Cys_108_ and Cys_112_) are predicted to form a double disulfide bridge (Figure 8b). A similar double disulfide bridge is predicted in the PCS “E” form of *T. pseudonana* between Cys_102_ and Cys_125_ and between Cys_103_ and Cys_121_ (Figure 8d), whereas in this diatom “N” PCS structure, a single disulfide bridge is predicted between Cys_164_ and Cys_184_ (Figure 8c). The cysteine arrangement is more complex in the PCS “D” structure, in which a single disulfide bond is formed between Cys_128_ and Cys_130_, but this is surrounded by a cysteine cluster involving Cys_18_, Cys_152_ and Cys_339_ (Figure 8e).

### 2.5. Domain Analysis

The PCS proteins of the selected model organisms were analyzed by using the MEME tool [53] to individuate the 15 most conserved motifs characterizing PCS sequences of different evolutive taxa.

**Figure 9:**
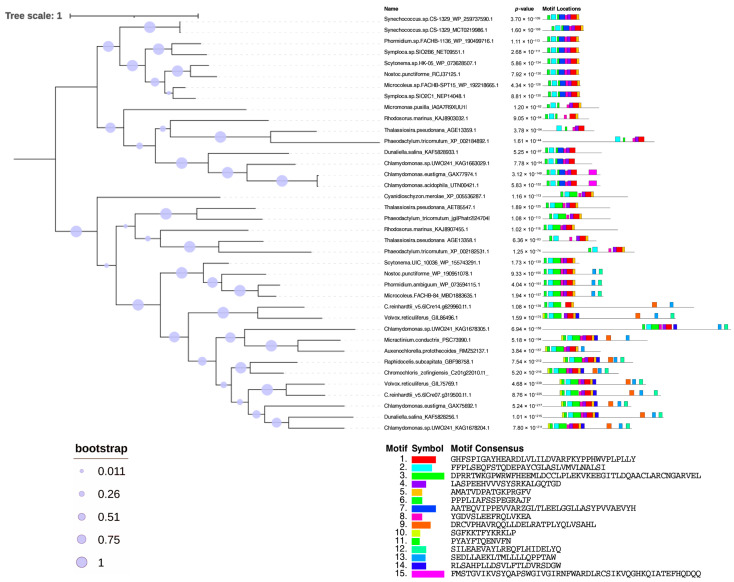
Phylogenetic tree showing 38 protein sequences of PCSs from 6 cyanobacteria and 15 eukaryotic algae belonging to different taxa and chosen as models (Table 1). The tree was constructed by MEGA11 with the ML method, and the bootstrap consensus tree was generated with 1000 replicates. The bootstrap percentage is represented by circles on each branch. The block diagram representation of the most conserved 15 motifs in the PCS protein sequences obtained with the MEME tool [53]. The catalytic domains are distributed closely together in the *N*-terminal domain. The less conserved *C*-terminal domain is present only in the “E” PCS sequences belonging to green algae and cyanobacteria closely related to them (the lowest sub-branch of the tree).

The alignment (shown in Figure 6) gave rise to a tree clearly divided into two branches, including 16 sequences belonging to the previously described group 1 (“N”) and 22 sequences belonging to group 2 (“E/D”), and both branches contain both cyanobacterial and eukaryotic sequences. While the “N” PCSs are clearly divided into prokaryotic and eukaryotic sequences (Figure 9), suggesting a separate evolution of these proteins in the two kingdoms, in the second branch, the sequences are divided between red algae/diatoms and cyanobacteria/green algae, suggesting that the phylogenetic history of this isoform is more closely related to the endosymbionts that gave rise to the different evolutionary lineages and that most likely those of the ancestors of red algae were not present among the retrieved sequences.

As already reported [45], the *N*-terminal domain is strictly conserved in a broad range of organisms, while the *C*-terminal domain is very variable. Most of the conserved motifs (see Figure 9 for their sequences) are indeed strictly grouped in the *N*-terminal protein domain; among them, there are motifs 1 and 2 containing the His and Asp (H-D) and the Cys (C) residues of the catalytic triad, respectively. Other largely conserved motifs are motif 4, absent only in the “N” form of *R. marinus* (KAJ8903032.1) and in *C. reinhardtii*_v5.6|Cre14.g629960.t1.1; motif 6, shared by all the sequences with the exception of the two diatoms “N” forms; and motif 8, not detected in *C. reinhardtii*_v5.6|Cre14.g629960.t1.1 and *V. reticuliferus*_GIL86496.1. Shared by 31 out of 36 PCS sequences is motif 5, which is lacking in the sequences of the eukaryotic algae of group 1. The remaining motifs are instead characteristic of more restricted sequence groups. Motif 11 was found only in the sequences of group 1, whereas motif 7 is restricted to the cyanobacteria and Chlamydomonadales of group 1 but was not detected in the Mamiellales (*Micromonas*), red algae (*R. marinus*) or diatoms (*T. pseudonana* and *P. tricornutum*) belonging to the same “N” group, indicating different evolutive lineages for these latter taxa. Peculiar of the “N” PCS form of *C. eustigma* (GAX77974.1) and *C. acidophila* (UTN00421.1) is 53 bp long motif 15; both these species are acidophilic green algae, and maybe, this domain confers particular properties to their PCS proteins. No analogous distinctive peculiarity was observed in the *C. eustigma* “E” PCS form (GAX75692.1) (not retrieved for *C. acidophila*). Motifs 3, 9, 10 and 12–14 were found only in the sequences of the “E” branch of group 2.

Further, 52 amino acid in length motif 3 contains the four conserved Cys residues described for land plants and is placed between the two motifs containing the catalytic residues (1 and 2). This motif is conserved in all the sequences of group 2 with the exception of the two diatom “D” sequences (*T. pseudonana* AGE13358.1 and *P. tricornutum* XP_002182531.1), in which the consensus motif was not recognized.

With the exception of the Trebouxiophyceae (*Auxenochlorella protothecoides* RMZ52137.1; *M. condutrix* PSC73990.1) and *C. reinhardtii*_v5.6|Cre14.g629960, motif 12 and motif 13 are present in all the “E” PCS cyanobacteria (with the exception of *Scytonema* WP_155743291.1) and in the closely related green algal sequences. Motifs 9, 10 and 14 are instead conserved exclusively in the *C*-terminal domain of the eukaryotic green algal sequences of group 2 (with the exceptions of *C. reinhardtii*_v5.6|Cre14.g629960 for motif 10 and *A. protothecoides* RMZ52137.1 for both the mentioned motifs).

## 3. Conclusions

To our knowledge, the analysis reported in the present paper is the first attempt to describe PCS sequences in eukaryotic algae and obtain more insights into their phylogenetic origin from cyanobacterial proteins. The analyzed sequences of PCSs and PCS-like proteins are divided into two branches that contain sequences corresponding to those previously described for the “half PCS sequences” of prokaryotes (considered primitive PCSs) and to the sequences described for higher plants. However, surprisingly, compared with what was reported in the previous literature, both prokaryotic and eukaryotic sequences were found in both branches. Diatoms, red algae, cyanobacteria and the extremophilic Chlamydomonadales possess both group 1 (“N”) and group 2 (“E”) forms. Cyanobacterial proteins, regardless of whether they belong to group 1 or 2, have a higher aliphatic index than eukaryotic proteins, which should confer them greater thermostability. Greater stability also appears to characterize group 1 (“N”) proteins, which could explain their presence in marine eukaryotic or extremophilic algae, which also possess a group 2 (“E”) PCS, suggesting that “N” isoforms are important in responding to particular environments adaptations. It needs to be clarified whether the two isoforms described represent proteins with different functions or activated under different cellular conditions. The “E” sequences share the features for being post-translationally regulated by phosphorylation, while the N ones do not. The latter, therefore, would not have the ability to form the pocket for the second substrate and thus to synthesize phytochelatins at a high degree of polymerization, as described for *Nostoc* NsPCS. The existence of N and E forms in cyanobacteria and in eukaryotic algae leads us to rethink what in previous works was indicated as the difference between cyanobacterial and eukaryotic PCSs. It is, therefore, likely that the previously found differences between the NsPCS of *Nostoc* and the AtPCS1 of *Arabidopsis* are not attributable to differences between prokaryotic and eukaryotic sequences, but rather to differences between proteins with different functions. Our results pave the way towards further biochemical analyses necessary to verify the involvement of the different PCS forms identified in response to cellular needs or to environmental stresses.

## 4. Materials and Methods

### 4.1. Phylogenetic Analysis

Evolutionary history was inferred by using the maximum likelihood method and the JTT matrix-based model [54] and conducted by using MEGA 11 software [55]. The initial tree(s) for the heuristic search was obtained automatically by applying the Neighbor-Join and BioNJ algorithms to a matrix of pairwise distances estimated by using the JTT model and then selecting the topology with the superior log likelihood value. The percentage of trees in which the associated taxa clustered together was calculated through the bootstrap test using 1000 replicates. The alignments were performed by using ClustalW. Tree representation was modified through iTOL “interactive Tree Of Life” [56].

Two known *C. reinhardtii* PCSs, Cre07.g319500 and Cre14.g629960, were retrieved from the annotated genome of this alga [46,47] (available on Phytozome 13 [48] (https://phytozome-next.jgi.doe.gov/, accessed on 20 October 2023)). The sequence of Cre07.g319500 was used as query against the NCBI database (www.ncbi.nlm.nih.gov/BLAST, last accessed on 26 March 2024) for proteomic accession through BlastP analysis [57]. Searches were performed across the different taxa present in the database. The GenBank accessions of the sequences used for the analysis are reported in Table S1. The consensus patterns in these candidate sequences were checked with PROSITE (https://prosite.expasy.org/scanprosite/, accessed on 30 October 2023) with the objective of including sequences of true PCSs, given that many analyzed genomes have not been completely annotated.

### 4.2. Sequence Analyses of PCSs

The retrieved sequences were aligned by ClustalX 2.0 [58] and MEGA 11 software [55] and visualized through Genedoc [59]. Sequence homology analyses were performed by using Blastp (www.ncbi.nlm.nih.gov/BLAST, last accessed on 26 March 2024), ClustalX (www.clustal.org/clustal2/, accessed on 26 March 2024) and WebLogo [60,61].

The physicochemical properties of the PCS proteins were analyzed with the ProtParam tool [50]. Statistical analysis of each parameter was performed by grouping the sequences on the basis of their belonging to group 1 or group 2, individuated by the phylogenetic tree, or their belonging to cyanobacteria or eukaryotic algae. The significance of the observed differences was checked through Student’s *t*-test by comparing group 1 vs. group 2 and cyanobacteria vs. eukaryotic algae after checking the normal distribution (Shapiro–Wilk test) and variance homogeneity of the data (Levene test).

### 4.3. Structural Analysis

The analysis of the structures of the active site and the residues potentially involved in regulation through phosphorylation, as well as the different cysteine arrangements of selected PCS sequences, was performed on predicted models found in the AlphaFold Protein Structure Database, an extensive database of high-accuracy protein-structure predictions [51,52].

### 4.4. Motif Analysis

The search for conserved motifs shared by the PCS proteins of the different cyanobacterial and algal taxa was carried out by the online web tool Multiple Em for Motif Elicitation (MEME) [53]. MEME represents motifs as position-dependent letter-probability matrices that describe the probability of each possible letter at each position in the pattern. Individual MEME motifs do not contain gaps [53]. All parameters were set to default except for max number of motifs to find and min/max width of motifs, which were set to 15 and 6–50, respectively. In this analysis, we included the NsPCS AD1928 of *Nostoc* sp. PCC7_120_ as the reference for prokaryotic “half PCS sequences” [20] and the Cre07.g319500 of *C. reinhardtii* sequences for eukaryotic algal PCSs. The Cre07.g319500 sequence was selected following a comparison with the AtPCS1 of *A. thaliana* (GenBank: OAO95078.1), sharing many features with the latter, including the *N*-terminal conserved region, which corresponded to the phytochelatin domain (EC 2.3.2.15), and a *C*-terminal variable region rich in Cys residues. Furthermore, it contains the four conserved Cys described in land plant PCSs [20,21].

## Figures and Tables

**Table 1:** Putative phytochelatin synthase (PCS) in 6 cyanobacterial and 15 eukaryotic algal species belonging to different taxa and their gene/protein features.

Order	Species	Accession No.	Exon No.	PCS Form °	Length (aa)	MW (kDa)	C-H Distance (aa)	H-D Distance (aa)	Cys Residues	Isoelectric Point	% Neg of Residues	% Pos of Residues	Instability Index *	Aliphatic Index	Habitat
Cyanobacteria															
Nostocales	*N. punctiforme* NIES-2108	RCJ37125.1	1	QN**N**QAYC	243	27.37	112	17	2	9.18	9.05	11.52	**31.78**	82.59	S
	*N. punctiforme*	WP_190951078.1	1	QSEPAFC	397	45.30	106	17	11	5.55	9.57	7.55	49.11	97.2	S
	*Scytonema* sp. HK-05	WP_073628507.1	1	QN**N**QAYC	245	27.48	112	17	2	9.47	7.34	10.61	**38.87**	89.1	S
	*Scytonema* sp. UIC_10036	WP_155743291.1	1	QSEPAYC	243	27.43	106	17	6	6.59	10.69	10.28	53.02	83.91	FW
Oscillatoriales	*Microcoleus* sp. FACHB-SPT15	WP_192218665.1	1	QK**N**QAYC	251	27.66	111	17	1	5.25	9.96	7.17	44.67	102.91	S
	*Microcoleus* sp. FACHB-84	MBD1883635.1	1	QAEPAFC	400	45.08	106	17	8	5.56	11.5	8.5	54.38	96.5	S
	*Phormidium* sp. FACHB-1136	WP_190499716.1	1	QI**N**QAFC	246	27.05	110	17	2	4.61	10.97	6.09	**38.82**	105.53	FW/M
	*P. ambiguum*	WP_073594115.1	1	QAEPAFC	394	45.12	106	17	10	5.59	10.65	8.12	54.85	103.43	FW/M
Coleofasciculales	*Symploca* sp. SIO2B6	NET09551.1	1	QQ**N**PAFC	245	26.77	110	17	1	4.78	9.79	5.71	41.47	106.78	M
	*Symploca* sp. SIO2C1	NEP14048.1	1	QR**N**GAYC	248	27.96	111	17	2	6.34	9.67	9.27	42.83	101.37	M
Synechococcales	*Synechococcus* sp. CS-1329	WP_259737590.1	1	QA**N**LAYC	244	26.27	112	17	2	9.55	6.55	8.61	43.23	109.63	FW/S
	*Synechococcus* sp. CS-1329	MCT0219986.1	1	QA**N**LAYC	271	29.26	112	17	3	9.86	6.27	9.22	44.05	108.08	FW/S
Algae															
Cyanidiales	*C. merolae*	XP_005536287.1	1	QSEPAFC	560	61.53	118	17	18	8.52	10.18	11.25	52.40	85.64	FW
Stylonematales	*R. marinus*	KAJ8903032.1	2	QV**N**QAFC	306	33.45	119	17	5	5.78	10.78	9.47	**31.04**	90.42	M
	*R. marinus*	KAJ8907455.1	5	QSEPAYC	495	55.36	103	17	13	5.12	15.55	11.92	54.53	77.76	M
Bacillariales	*P. tricornutum*	XP_002184892.1	2	QI**N**QAYC	735	82.10	136	17	5	5.72	11.84	8.71	41	70.82	M
	*P. tricornutum*	Phatr_24704	1	QSEPAYC	447	50.56	114	17	11	5.75	11.85	10.74	43.64	82.91	M
	*P. tricornutum*	XP_002182531.1	3	QSDPAYC	604	67.08	140	17	14	9.74	7.95	11.59	51.76	75.38	M
	*T. pseudonana*	AGE13359.1	2	QI**N**QAYC	340	37.51	127	17	5	5.41	11.18	9.41	**38.34**	77.74	M
	*T. pseudonana*	AGE13358.1	2	QSDPAYC	354	39.88	155	17	10	7.15	11.3	11.3	52.39	68.5	M
	*T. pseudonana*	Thaps_257216	2	QPEPAYC	444	49.85	112	17	10	5.62	11.94	10.81	47.65	83.2	M
Mamiellales	*M. pusilla*	A0A7R9XUU1	2	QR**N**QAFC	375	40.34	128	17	5	5.47	13.07	10.13	**38.64**	88.29	M
Chlamydomonadales	*D. salina*	KAF5828933.1	9	QI**N**SAFC	389	42.32	118	17	2	4.95	10.03	6.17	**38.18**	86.74	M/FW
	*D. salina*	KAF5828256.1	12	QDEPAFC	793	83.83	105	17	16	6.34	8.45	7.06	53.1	61.02	M/FW
	*C. reinhardtii* v5.6	Cre14.g629960.t1.1	8	QDEPAFC	994	96.73	94	17	32	5.78	7.75	6.34	56.1	75.77	FW
	*C. reinhardtii* v5.6	Cre07.g319500.t1.1	11	QEEPQYC	776	78.70	107	17	17	6.66	8.25	7.86	41.71	78.04	FW
	*C. eustigma*	GAX77974.1	6	QV**N**DAYC	381	42.28	118	17	7	5.14	11.02	7.87	**33.59**	90.6	Acidic FW
	*C. eustigma*	GAX75692.1	10	QDEPAFC	582	63.97	105	17	18	6.76	9.45	9.11	54.87	79.3	Acidic FW
	*C. acidophila*	UTN00421.1	5	QV**N**DAYC	381	42.22	118	17	7	5.15	11.28	8.13	**34.14**	90.08	Acidic FW
	*Chlamydomonas* sp. UWO241	KAG1663029.1	8	QV**N**GAFC	326	34.02	117	17	1	5.13	9.20	6.44	**29.83**	90.71	M
	*Chlamydomonas* sp. UWO241	KAG1678305.1	15	QAEPAFC	1236	126.85	112	17	28	8.34	9.39	9.95	49.89	70.91	M
	*Chlamydomonas* sp. UWO241	KAG1678204.1	8	QDEPAFC	586	60.78	106	17	10	6.4	8.87	7.85	44.85	77.53	M
	*V. reticuliferus*	GIL86496.1	9	QEEPQYC	873	92.36	105	17	33	5.55	10.65	8.82	52.53	78.03	FW
	*V. reticuliferus*	GIL75769.1	10	QDEPAFC	679	72.85	105	17	15	6.56	9.13	8.54	53.03	79.87	FW
Chlorellales	*A. protothecoides*	RMZ52137.1	4	QDEPAFC	381	41.40	105	17	9	5.44	12.6	9.45	53.9	83.07	Acidic FW
	*M. condutrix*	PSC73990.1	10	QDEPAYC	689	74.73	105	17	13	6.25	10.45	9.58	64.51	68.4	FW
Sphaeropleales	*R. subcapitata*	GBF98758.1	6	QDEPAFC	594	61.77	105	17	15	6.68	8.92	8.42	45.04	79.04	FW
	*C. zofingiensis*	Cz01g22010.t1	7	QDEPAFC	500	54.77	105	17	17	6.21	10	8.2	49.95	78.92	FW

° In bold and underlined, the Asn residue of the “N” isoform; * in bold and underlined the proteins considered stable (index value below 40); % Neg of res. = (D + E)/tot × 100; % Pos of res. = (R + K)/tot × 100; S, soil; FW, fresh water; M, marine.

## Data Availability

Data are contained within the article and Supplementary Materials.

## Supplementary Materials

The following supporting information can be downloaded at: https://www.mdpi.com/article/10.3390/plants13152165/s1, Figure S1: Sequence alignment between PCS of *C. rehinardtii* (Cre07.g319500) and two sequence models of eukaryotic (AtPCS1 from *A. thaliana*) and prokaryotic PCSs (NsPCS from *Nostoc* sp. PCC7_120_), Figure S2: PCS distribution in cyanobacterial orders, Figure S3: Partial representation of multiple-sequence alignment of PCS proteins showing the discriminant residues N, E and D, Table S1: Sequences used for phylogenetic analyses.
