# Supplementary material for: Phytochelatin Synthase: An In Silico Comparative Analysis in Cyanobacteria and Eukaryotic Microalgae
Source: Plants (Basel). 2024 Aug 5;13(15):2165. doi: 10.3390/plants13152165 (PMC11314372; doi:10.3390/plants13152165)
Supplement: Supplementary file 1 [file plants-13-02165-s001.zip › Supplementary Figures.pdf]

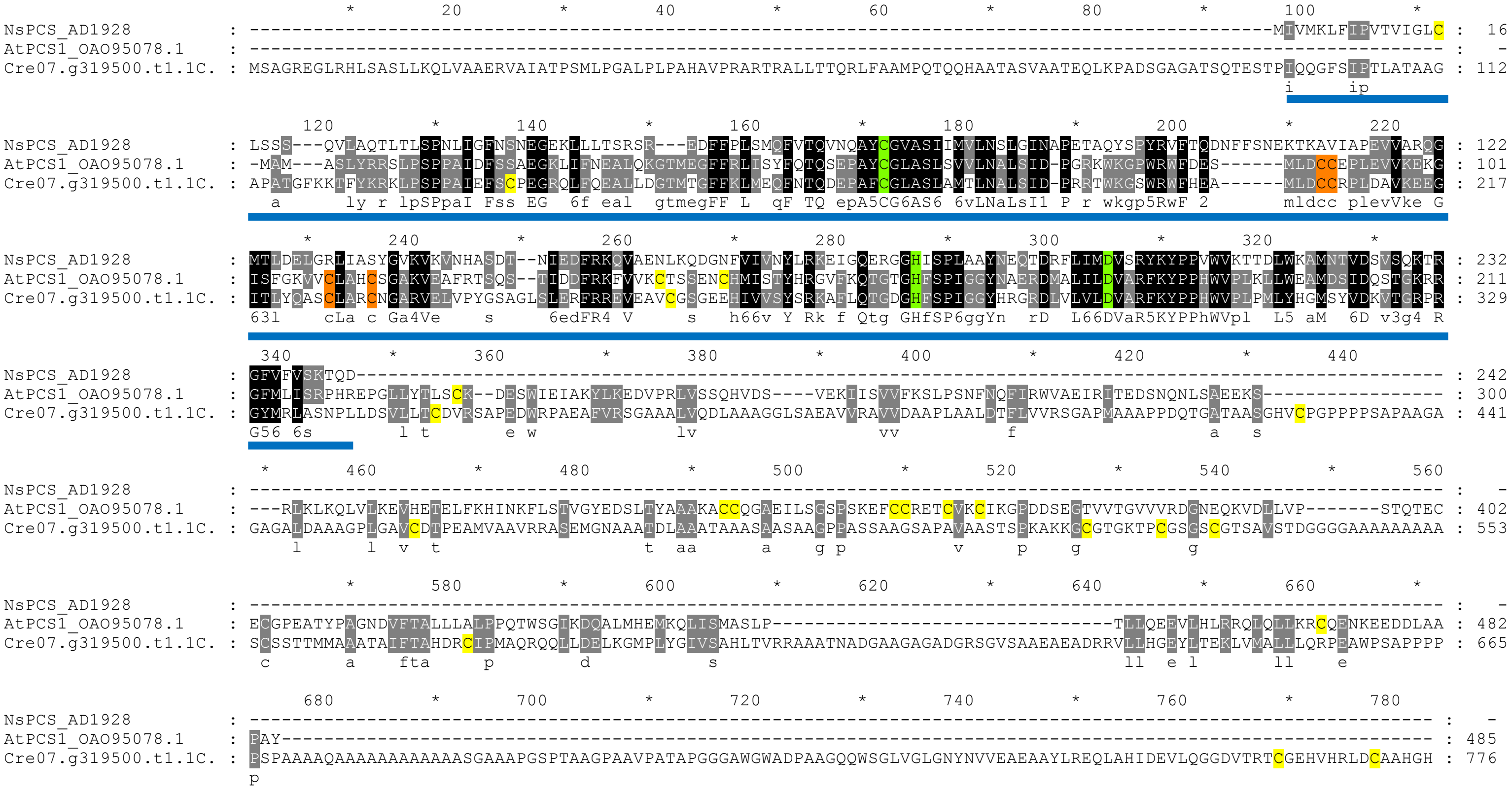

Figure S1: Sequence alignment between PCS of *C. rehinardtii* (Cre07.g319500) and two sequences model of eukaryotic (AtPCS1 from *A. thaliana*) and prokaryotic PCS (NsPCS from *Nostoc* sp. PCC7$_{120}$). Alignment was conducted with ClustalW; identical and similar residues are shaded in black and gray and consensus sequence is shown below alignment. Catalytic triad residues (Cys, His and Asp) are highlighted in green and cysteines in yellow. In orange the four conserved cysteines described in land plant PCS sequences. Underlined in blue the phytochelatin domain (EC 2.3.2.15).

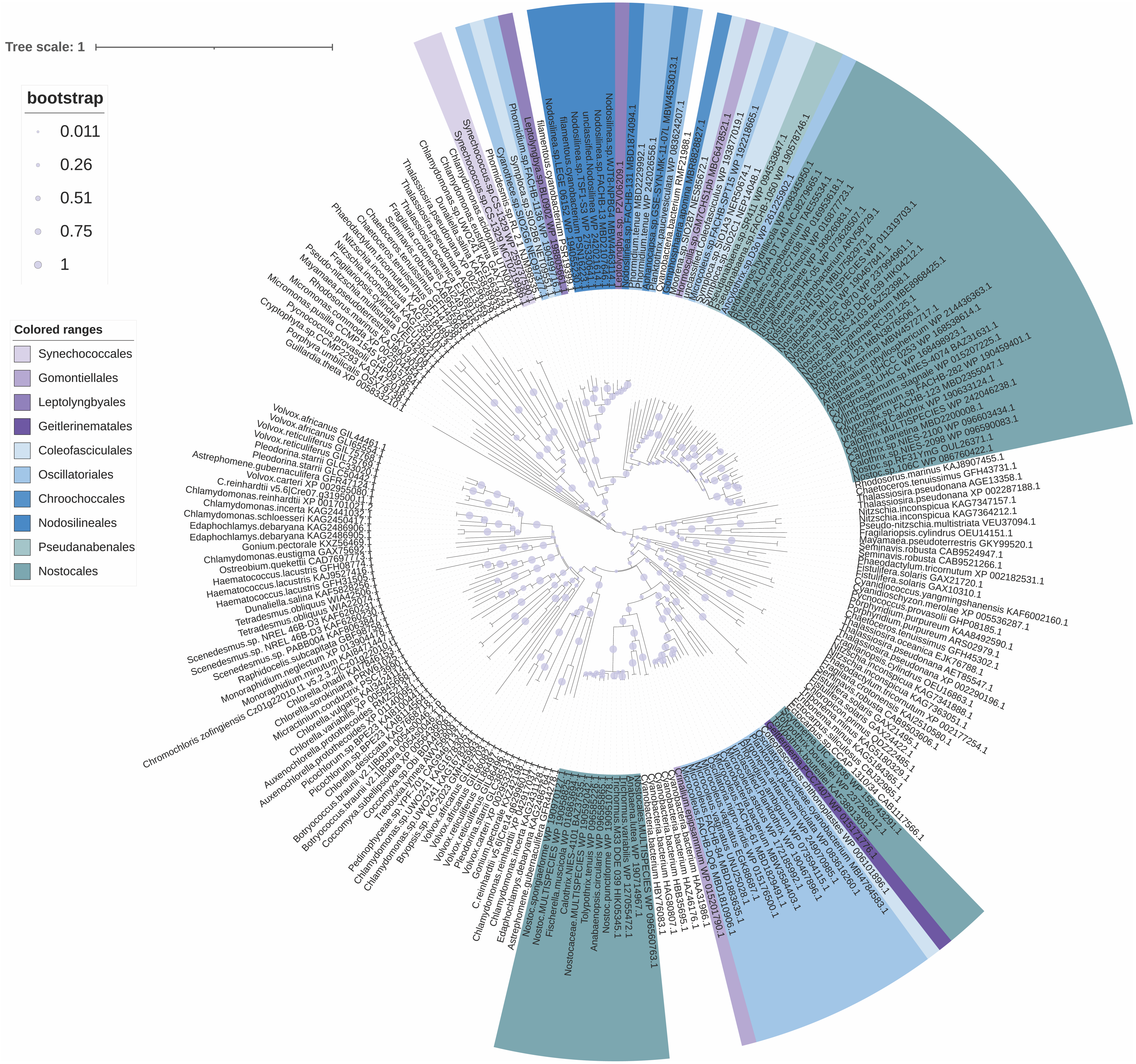

Figure S2: PCS distribution in cyanobacteria orders. The maximum likelihood tree is shown. The percentage of trees in which the associated taxa clustered together is shown below the branches. The tree is drawn to scale, with branch lengths measured in the number of substitutions per site. This analysis involved 220 amino acid sequences (Table S1) for a total of 3208 positions in the final dataset. The current tree is the same tree represented in Figure 1 except for coloration: different colors in the range of blue-violet represent different cyanobacterial orders as described by the legend on the left.

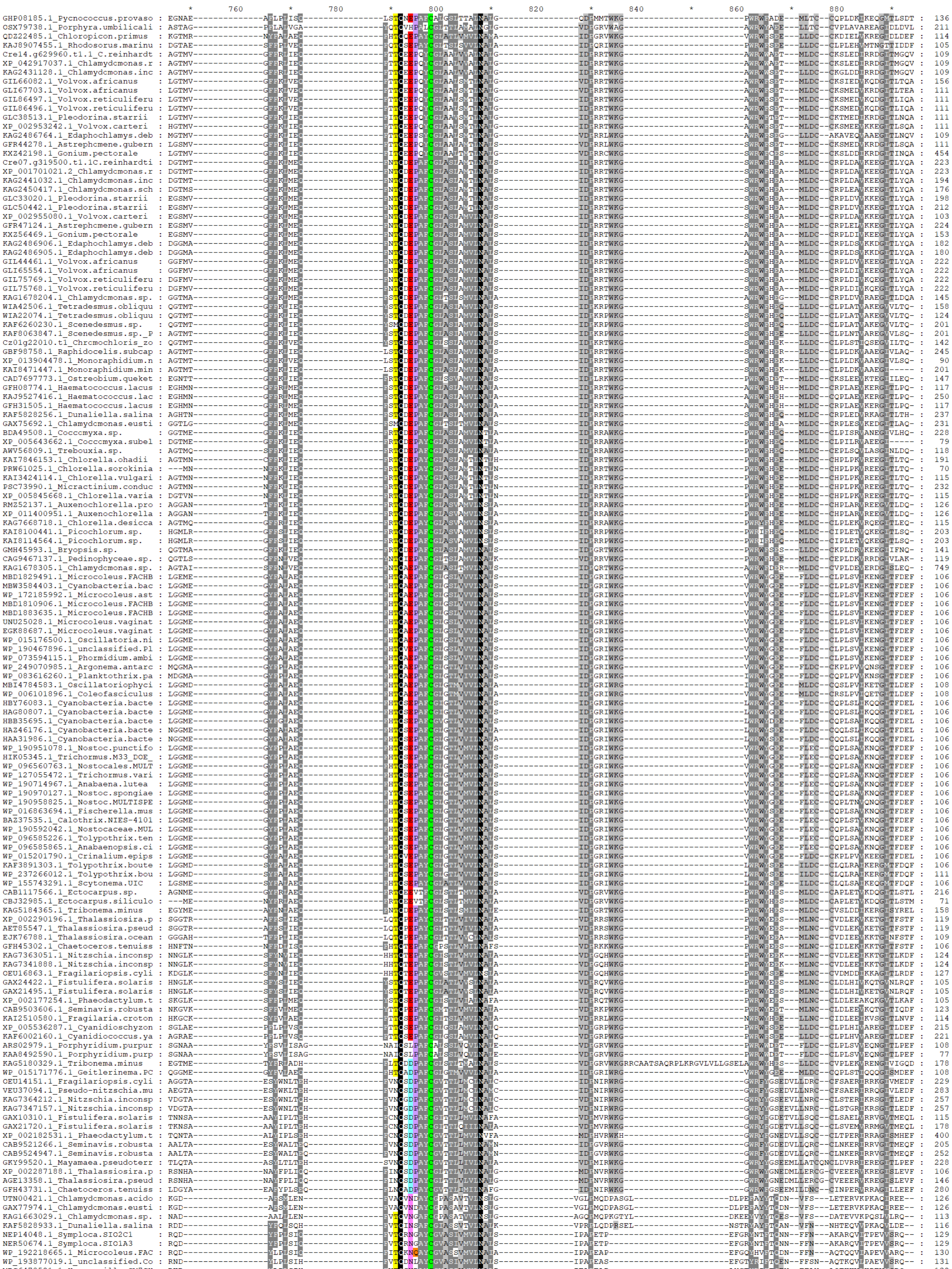

Figure S3: Partial representation of multiple sequence alignment of PCS proteins showing the discriminant residues N, E, D. Partial representation of multiple sequence alignment of PCS protein sequences referred in Table S1. Alignment was conducted with ClustalW; identical and similar residues are shaded in black and gray and consensus sequence is shown below alignment. Sequences of group 1 are characterized by an asparagine residue (N, magenta), often followed by a glutamine (Q, orange) four amino acids upstream the catalytic cysteine (C, green). In the sequences of the group 2 the asparagine residue is substituted by residue of glutamic acid (E, red), or by an aspartic acid (D, pale blue) in a sub-group of diatoms, followed by a proline (P, lilac). In yellow the threonine (T) residue possible target of phosphorylation.
